# Pediatric whole-body magnetic resonance imaging: comparison of STIR and T2 Dixon sequences in the detection and grading of high signal bone marrow changes

**DOI:** 10.1007/s00330-023-09413-6

**Published:** 2023-01-26

**Authors:** P. Zadig, E. von Brandis, L. S. Ording Müller, L. Tanturri de Horatio, K. Rosendahl, D. F. M. Avenarius

**Affiliations:** 1 Department of Radiology, University Hospital of North-Norway, Tromsø, Norway; 2 Department of Clinical Medicine, UiT, The Arctic University of Norway, Tromsø, Norway; 3 Division of Radiology and Nuclear Medicine, Oslo University Hospital, Oslo, Norway; 4 Department of Pediatric Radiology, Ospedale Pediatrico Bambino Gesù, Rome, Italy

**Keywords:** Pediatrics, MR imaging, Bone marrow, Imaging sequences, Whole-body imaging

## Abstract

**Objectives:**

To compare short time inversion recovery (STIR) and T2 Dixon in the detection and grading of high signal intensity areas in bone marrow on whole-body MRI in healthy children.

**Methods:**

Prospective study, including whole-body 1.5-T MRIs from 77 healthy children. Two experienced radiologists in consensus identified and graded areas of high bone marrow signal on STIR and T2-weighted (T2W) turbo spin echo (TSE) Dixon images (presence, extension) in two different sessions at an interval of at least 3 weeks. In a third session, a third observer joined the two readers for an additional consensus reading with all sequences available (substitute gold standard).

**Results:**

Four hundred ninety of 545 (89.9%) high signal areas were visible on both sequences, while 27 (5.0%) were visible on STIR only and 28 (5.1%) on T2W Dixon only. Twenty-four of 27 (89%) lesions seen on STIR only, and 25/28 (89%) seen on T2W Dixon only, were graded as mildly increased signal intensity. The proportion of true positive high signal lesions was higher for the T2W Dixon images as compared to STIR (74.2% vs. 68.2%) (*p* = 0.029), while the proportion of false negatives was lower (25.9% vs. 31.7% (*p* = 0.035) for T2W Dixon and STIR, respectively). There was a moderate agreement between the T2W Dixon and STIR-based extension scores on a 0–4 scale, with a kappa of 0.45 (95% CI = 0.34–0.56).

**Conclusions:**

Most high signal bone marrow changes identified on a 1.5-T whole-body MRI were seen on both STIR and water-only T2W Dixon, underscoring the importance of using identical protocols when following bone-marrow signal changes over time.

**Key Points:**

• *Whole-body MRI is increasingly being used to diagnose and monitor diseases in children, such as chronic non-bacterial osteomyelitis, malignant/metastatic disease, and histiocytosis.*

• *Standardized and validated imaging protocols, as well as reference standards by age for the growing skeleton are lacking.*

• *Prospective single-center study showed that 90% of high signal bone marrow areas identified on a 1.5-T whole-body MRI in healthy children is seen on both STIR and water-only T2W Dixon, while 5% is seen on STIR only and 5% on T2W Dixon only.*

## Introduction

Whole-body MRI is increasingly being used to diagnose and monitor diseases, such as chronic non-bacterial osteomyelitis, malignant/metastatic disease, Langerhans cell histiocytosis, syndromes, and genetic predisposition with increased tumor risk, and more recently, juvenile idiopathic arthritis [1]. However, standardized and validated imaging protocols, as well as reference standards by age for the growing skeleton, are lacking. A recent systematic review showed that the typical whole-body MRI protocol in children includes fluid-sensitive sequences only, or fluid sensitive along with T1-weighted (T1W) sequences, but the technical settings varied considerably across institutions [2]. Of fluid sensitive sequences, all being fast spin echo or turbo spin echo sequences, a short time inversion recovery (STIR) was by far the most common, being well known for its robust fat suppression. Alternative techniques including T2-weighted (T2W) Dixon were used/mentioned in only two out of the 54 studies [2]. In contrast, the Dixon technique is well-established in adult radiology, with a variety of musculoskeletal applications [3].

Several techniques enable fat signal suppression, based on (1) difference in resonance frequency with water by means of frequency selective pulses: fat saturation (fat-sat) techniques; (2) phase contrast techniques, (3) short T1 relaxation time by means of inversion recovery sequences (STIR technique), (4) Dixon method, and (5) hybrid techniques combining several of these fat suppression techniques such as spectral pre-saturation with inversion recovery [4]. The different fat suppression techniques have their advantages and disadvantages; while STIR is less sensitive to B0- and B1-heterogeneity and provides additional T1 and T2 contrast, it has a lower signal to noise due to the inversion delay. Using the Dixon technique, on the other hand, provides a better signal-to-noise ratio (SNR), and generates “water-only,” out of phase, in phase, and “fat-only” images simultaneously, and provides additional information on anatomy and water/fat content. The sequence is hampered with a specific artifact, the Dixon swap; however, recent software updates have reduced this problem.

In adults, the T2W Dixon and STIR techniques have been compared in a few studies showing diverging results [5, 6], while no such studies have been published for children. We have previously shown that grading high signal bone marrow intensity on a 0–2 scale performs well based on T2W Dixon images [7]. The purpose of this study was to compare T2W Dixon and STIR in the detection and grading of high signal intensity bone marrow changes at 1.5 T, on whole-body MRI in healthy children.

## Materials and methods

### Subjects

The study is part of a prospective multicenter study including 196 healthy children. The present sub-study includes 77 whole-body MRI examinations from a subgroup of 77 healthy children who had both STIR and T2W turbo spin echo (TSE) Dixon images taken as part of the WBMRI protocol in one of the participating centers. The volunteers were recruited via emails, direct invitations, or announcements on social media during June 2018 to March 2020. All children were scanned for research purposes only. Excluded were individuals having contraindications to MRI, a history of cancer, current infection, chronic or systemic disease, metabolic or musculoskeletal disorder, or a symptomatic trauma within the past 4 weeks. Also excluded were individuals with musculoskeletal complaints impairing everyday activity and/or necessitating a consultation by a physician within the last 6 months. Self-reported sport activities and hours of physical exercise per week were registered, as was height, weight, and handedness. None of the participating individuals reported on disease or symptoms from the musculoskeletal system when contacted within 18 months after the first examination. Children with the most conspicuous nonspecific bone marrow hyperintensities were invited to undergo a dedicated follow-up MRI [8]. In addition, all participants were contacted within 18 months after the initial MRI scan to confirm that no musculoskeletal symptoms had occurred.

The study was approved by the Regional Ethics Committee (REK; no 2016/1696), and written informed consent was obtained from all the participants or their caregivers as appropriate.

### MR imaging acquisition

All the examinations were performed at the University Hospital of North Norway on a 1.5-T scanner (Philips Ingenia, release 2.3) equipped with phased array surface coils. A total of five contiguous coronal stacks from skull base to toes were performed. All 77 children had T2W TSE Dixon and STIR sequences taken, while 35 had additional coronal T1 and diffusion-weighted images. Image parameters are detailed in Table 1. The STIR sequence was performed at the end of the examination.

**Table 1:** Basic MRI parameters for the whole-body MRI, T2W TSE Dixon, and STIR sequences. There was no refocusing pulse for either of the sequences

Sequence	TR (ms)	TE (ms)	TI (ms)	NSA	Scan time (min)	Slice thickness/increase (mm)	Echo train length	Readout band width (Hz)	Field of view (mm)	Acquired voxel size (mm)
Coronal STIR	3500	80	160	1	3:16	3.5/4.2	27	312	400 × 500	0.9 × 0.9 × 3.5
Coronal T2W TSE Dixon	5100	100	na	1	3:16	3.5/4.2	20	293	400 × 500	0.9 × 0.9 × 3.5

### Image analysis

**Fig. 1:**
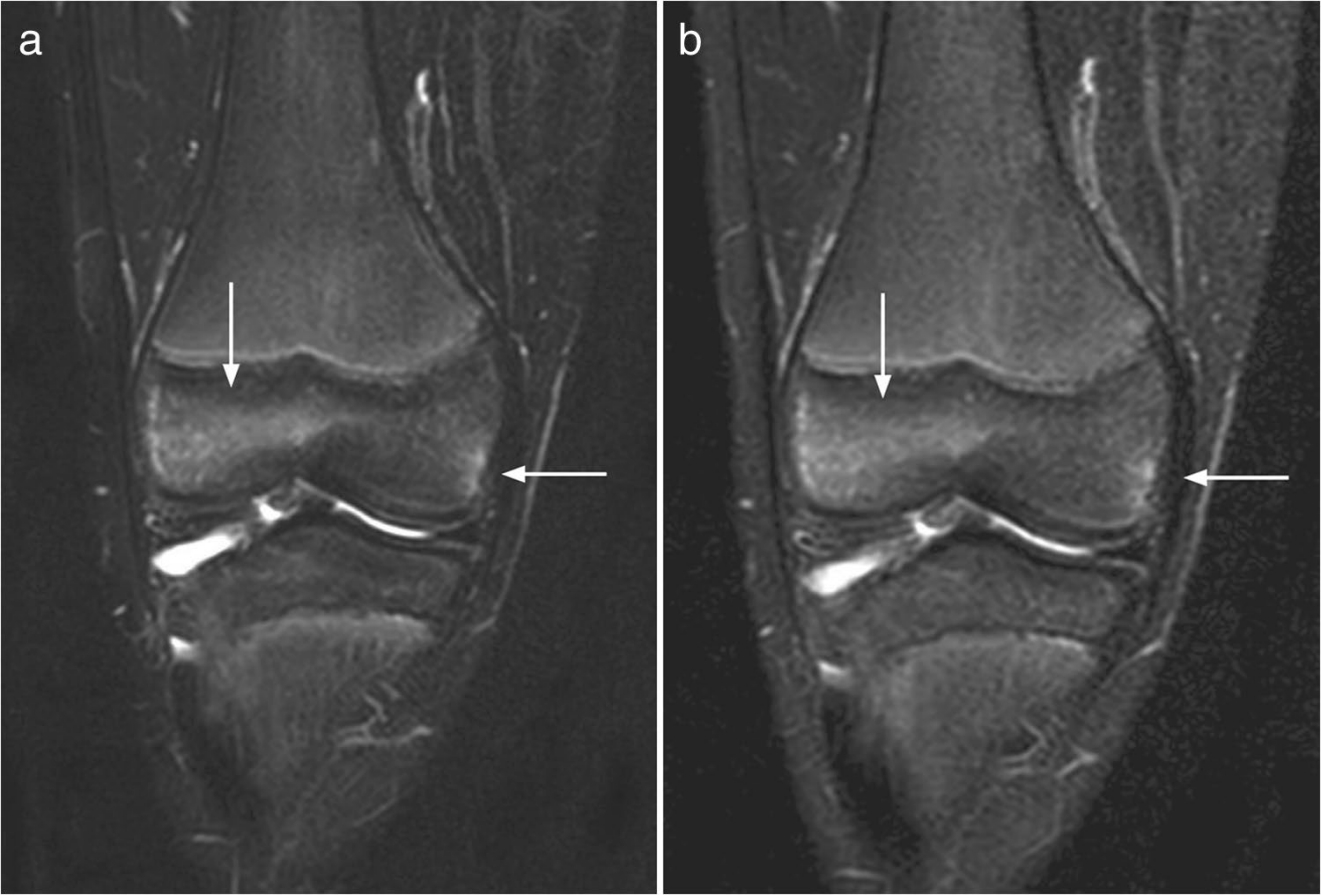
Coronal Dixon (**a**) and STIR (**b**) weighted images in a 10-year-old boy, with areas of grade 2 increased signal in the distal epiphysis of the femur (arrows), that are equally well visualized on both sequences

**Fig. 2:**
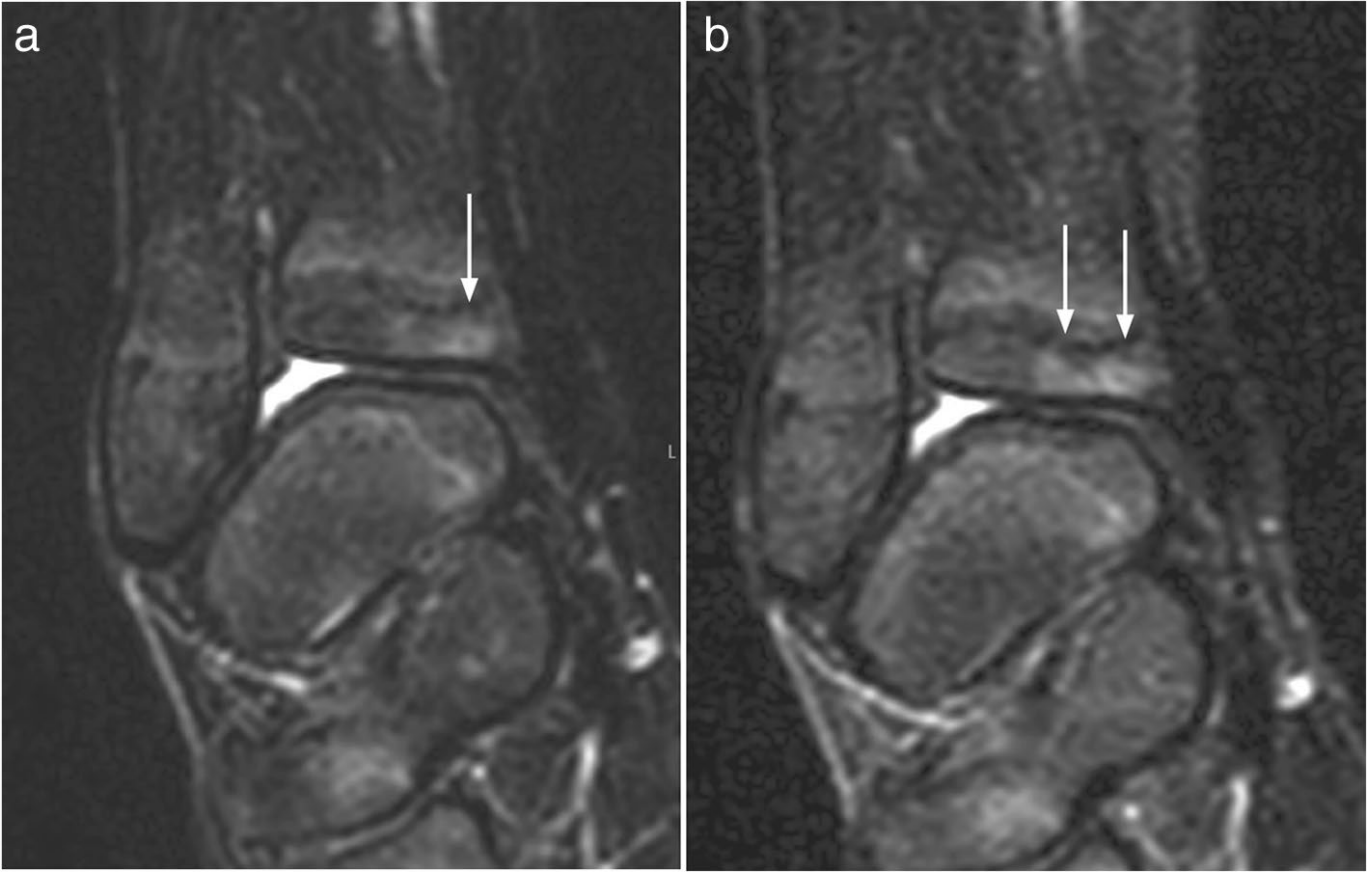
Coronal Dixon (**a**) and STIR (**b**) weighted images in a 10-year-old girl, showing different delineation of bone marrow signal in the distal tibia epiphysis (arrows), influencing both the perceived signal intensity and its extension

In a first session, two radiologists with 5 years (P.Z.) and 20 years (D.A.) of experience in MR imaging evaluated the coronal STIR images for the presence and extension of high signal bone marrow intensities, in consensus. After an interval of at least 3 weeks to avoid recall bias, the same readers evaluated the T2W TSE Dixon images, blinded for additional information, using the same Sectra viewing system (IDS7 PACS) and room-light settings. The high signal intensities were located (skull, spine, clavicle, scapula, sternum, upper arm, forearm, hand, pelvis, thigh, leg, and foot) as was area within the bone (epi-, meta, or diaphysis), and scored for conspicuity (intensity) on a 0–2 scale (0 = absent/no high signal area, 1 = mildly increased, 2 = moderately increased up to fluid-like signal). Except for the feet, extension of the high signal area was scored on a 0–4 scale (0 = absent/no high signal area, 1 ≤ 5%, and then increments of 1/3 of the subjectively perceived volume of bone segment) (Fig. 1, Fig. 2).

Areas of high signal were noted in an anatomical drawing on paper for each of the two scoring sessions. In a third session, a third observer (K.R., > 25 years of experience in pediatric MRI) joined the two readers for an additional consensus reading with all sequences available. This was considered a substitute gold standard. In a recent paper, we found that the grading system for signal intensity used in the present paper performed well, with good to very good intra- and interobserver agreement based on T2W Dixon images [7].

Locations/areas flawed by image artifacts or by too low SNR for image analysis were registered and excluded. Subcortical high signal lines/stripes extending from the diaphysis to the metaphysis, linear hyperintensity parallel to the physis, the presence of diffusely distributed hyperintensity signal in the metaphysis and diaphysis of long bones (“increased background signal”), vertical lines in the diaphysis (obvious vessels), speckled high signal in the wrist and feet (up to 5 mm), lines and dots (thin lines and dots up to 2 mm) in the epiphysis, and when relevant, the presence of focal periphyseal edema (FOPE) were registered but not further characterized.

### Statistical analysis

The number of high signal intensity lesions and conspicuity for each lesion as well as the number of excluded areas/localizations due to low SNR were tallied for each of the two sequences. Estimates of sensitivity and specificity (with 95% CI) were calculated for each sequence, by summating all false-positive, false negative, true positive, and true negative lesions. The consensus reading with all sequences available by three observers was considered the substitute gold standard. Differences in the proportions of true positives and false negatives between the two sequences were examined using chi-square test.

Grading of signal intensity by the two readers in consensus, based on T2W Dixon and STIR images, were displayed in a contingency table; McNemar test was used to test for systematic differences and Cohen’s kappa (linear weighted when > 2 categories) (with 95% confidence intervals (CIs)) and percentage absolute agreement were used to examine agreement. A kappa score of < 0.2 is considered poor, 0.21–0.40 fair, 0.41–0.60 moderate, 0.61–0.80 good, and 0.81–1.00 very good [9]. To test for differences in agreement between STIR and Dixon (yes/no) according to age, we grouped the children into four age groups: < 9 years, 9–12 years, 12–15 years, and > 15 years. A significance level of 0.05 was decided a priori, and all the reported *p* values are two-tailed. The analysis was performed using Predictive Analytics Software (SPSS) version 27.

## Results

**Fig. 3:**
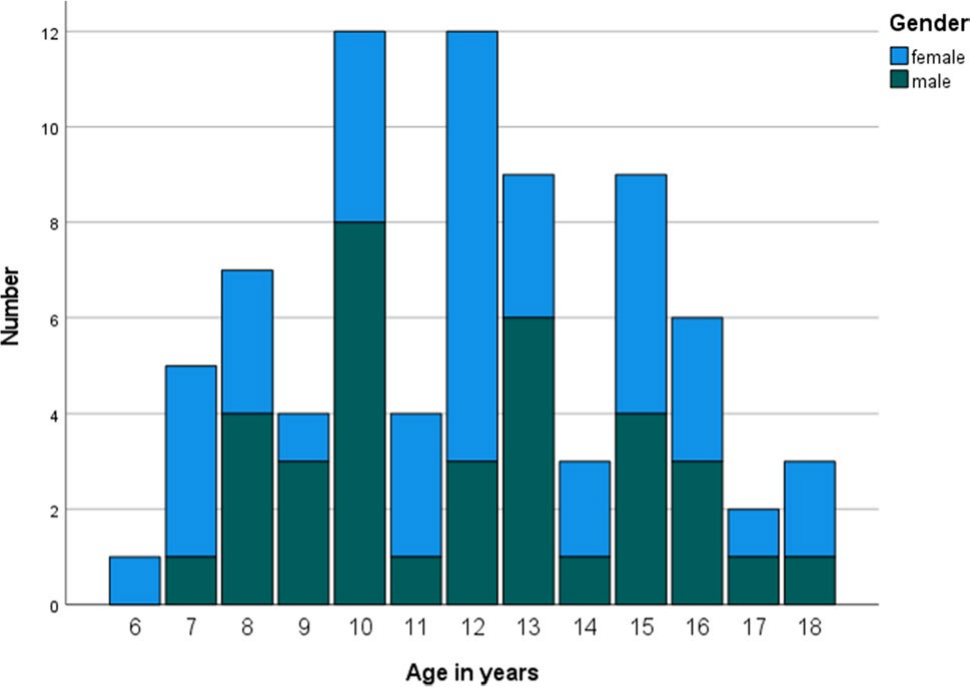
Distribution of age and gender for the 77 children and adolescents included in the study

A total of 77 whole-body MRI examinations in 77 healthy children (36 males) between 6 and 19 years (mean age 12 years (SD 3.1)) were included (Fig. 3). A total of 28,100 images were analyzed. All children had at least one area of high signal (median 6 areas, range 1–30). A total of 127/3311 (3.8%) anatomical areas were excluded due to artifacts or suboptimal SNR, of which 78 (61.4%) were based on STIR alone, 23 (18.1%) were based on both STIR and Dixon images, and 16 (12.6%) were based on Dixon alone. Except for two, the excluded areas were shoulder, elbow, hand, or foot.

A total of 568 lesions were identified and scored during the initial readings, of which 6 (1.1%) in the spine, 8 (1.4%) in the thoracic cage, 58 (10.2%) in the upper extremities, 41 (7.2%) in the pelvis, and 435 (76.6%) in the lower extremities. On the 3-reader consensus with all sequences and Dixon reconstructions available (substitute gold standard), 23/568 (4.1%) lesions (2 in the upper extremities and 21 in the pelvis/lower extremities) were judged insignificant, thus should not have been scored, leaving 545 “true” lesions for further analysis.

**Fig. 4:**
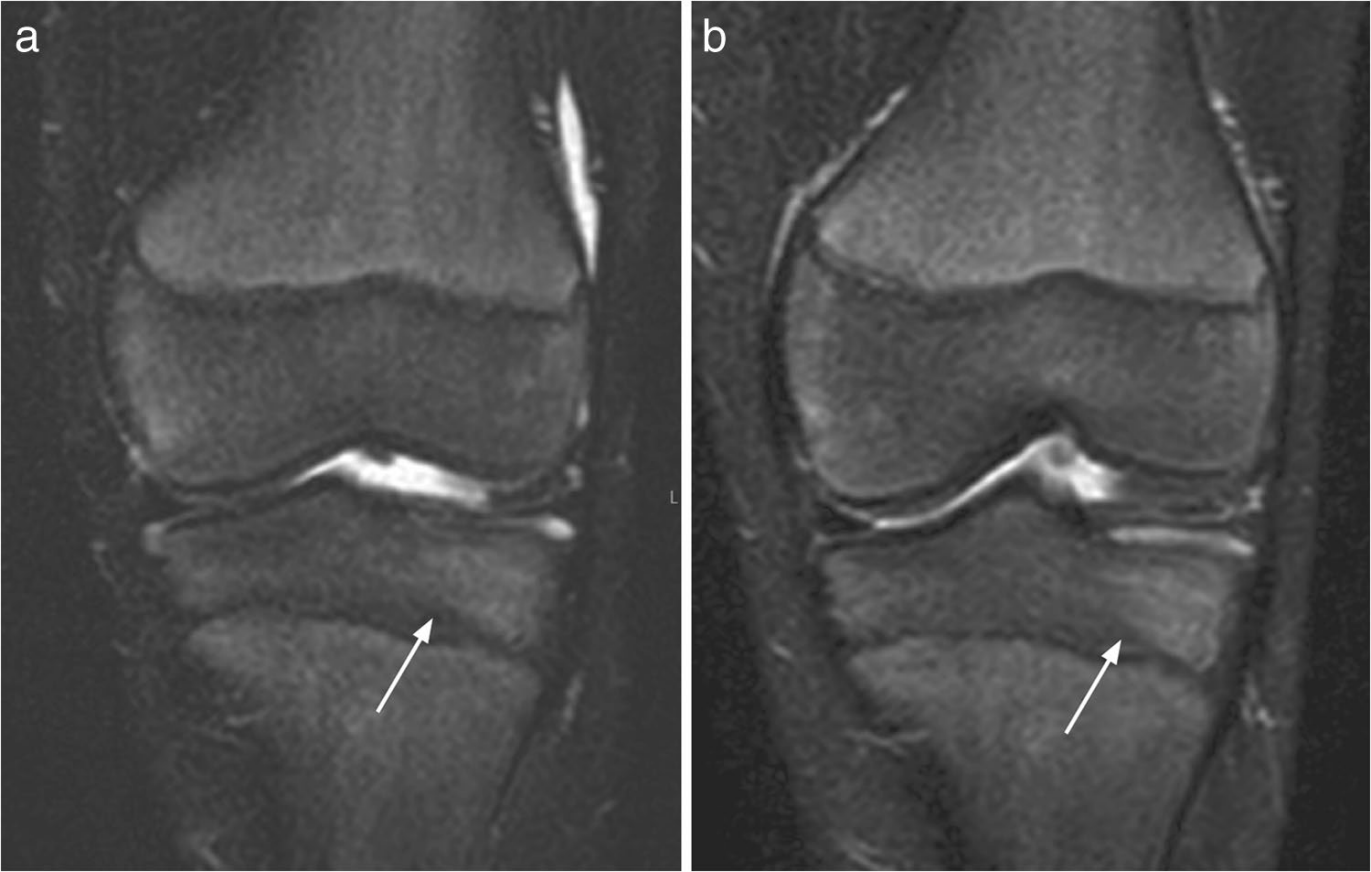
Coronal Dixon (**a**) and STIR (**b**) weighted images of the knee in a 12-year-old boy, showing subtle movement between scans, giving the impression of a sharper delineation of the grade 1 high signal seen in the proximal lateral tibia epiphysis on the STIR image (arrows)

**Fig. 5:**
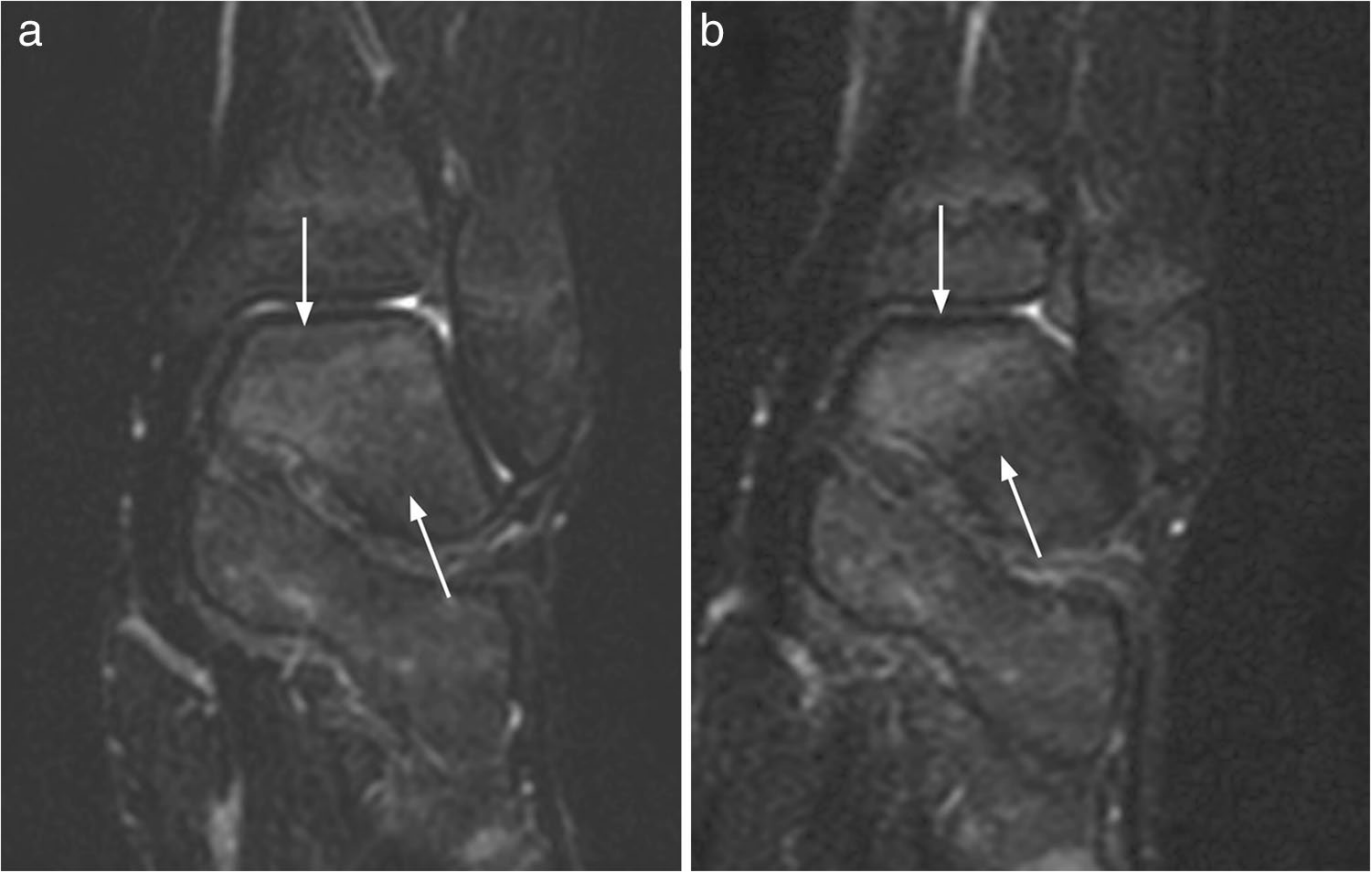
Coronal Dixon (**a**) and STIR (**b**) weighted images of the ankle in a 10-year-old boy, showing subtle movement between scans, which may explain the difference in scores given; mildly increased signal on Dixon and moderately increased signal on STIR

Based on the substitute gold standard, 490 of the 545 (89.9%) “true” lesions were visible on both sequences, while 27 (5.0%) were visible on STIR only and 28 (5.1%) were visible on T2W Dixon only (Table 2) (Fig. 4, Fig. 5). The rates of “true” lesions visible on both sequences ranged from 75% for the pelvis to 92.9% for the upper extremities (Table 2) while corresponding percentages for the axial skeleton and thoracic cage could not be estimated due to low numbers (*p* < 0.001).

**Table 2:** Detection of areas with increased bone marrow signal on WB MRI STIR and Dixon-weighted sequences (substitute gold standard; consensus between 3 observers), by localization, in 77 healthy children and adolescents aged 6–19 years

		Visibility			Total
		STIR only	Dixon only	Both (%)	
Localization	-Axial skeleton	0	1	5	6
	-Thoracic cage	3	2	3	8
	-Upper extremities	2	2	52 (92.9)	56
	-Pelvis	5	5	30 (75.0%)	40
	-Lower extremities	17	18	400 (92.0)	435
Total		27	28	490 (89.9)	545

**Fig. 6:**
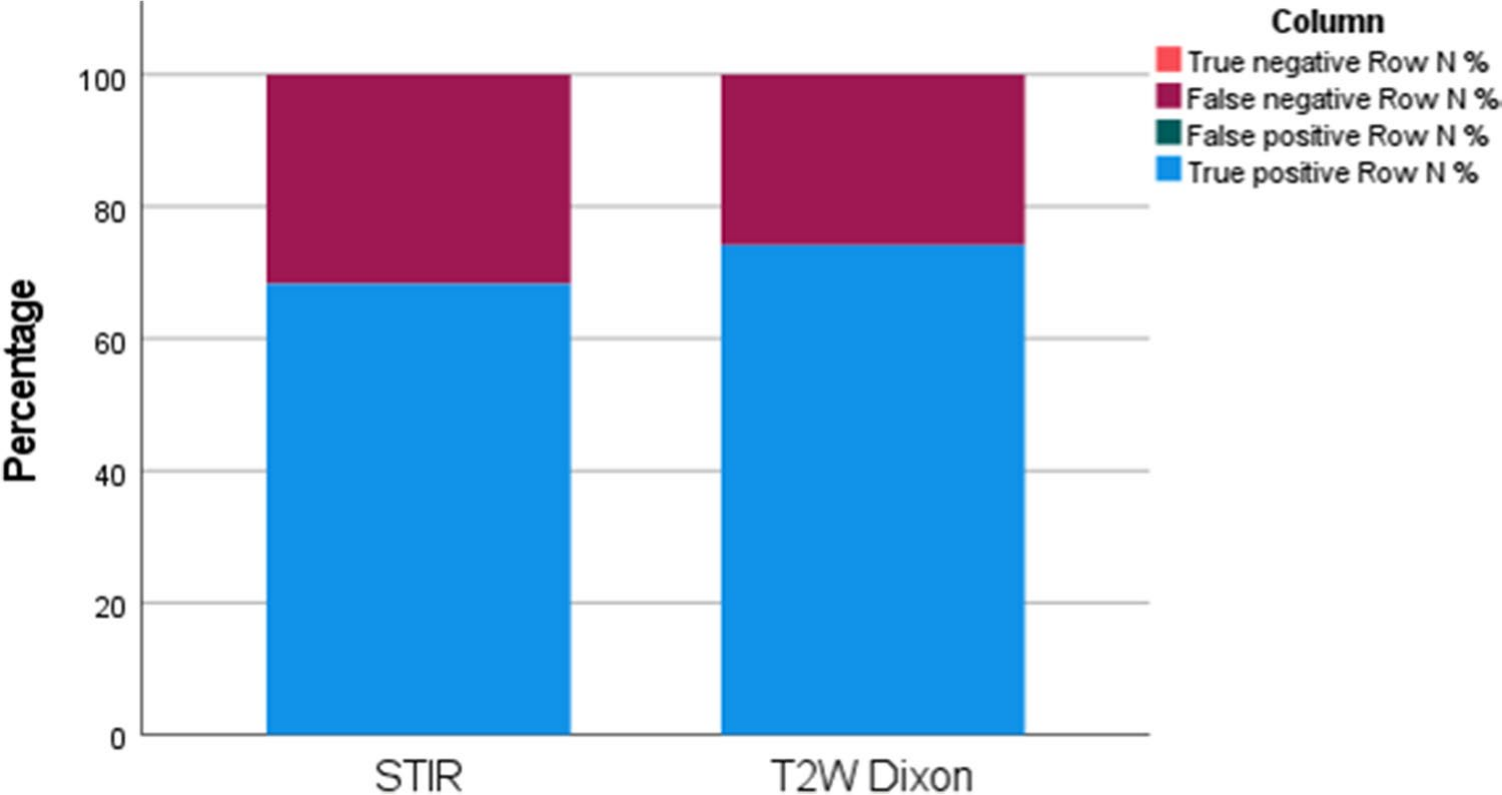
Stacked bar chart showing the proportion of true positive, false positive, false negative, and true negative high signal intensity areas for 545 lesions, as per the substitute gold standard

The proportion of true positive high signal lesions was higher for the T2W Dixon images as compared to STIR (74.2% (95% CI = 70.5–77.8%) vs. 68.2% (95% CI = 64.3–72.2%) (*p* = 0.029), while the proportion of false negatives was lower (25.9% (95% CI = 22.2–29.5%) vs. 31.7% (95% CI = 31.7–27.8%)) (*p* = 0.035) for T2W Dixon and STIR, respectively) (Fig. 6).

The initial agreement between T2W Dixon and STIR for grading of signal intensity, i.e., based on the 545 “true” lesions was poor, with a kappa value of − 0.02, increasing to 0.34 (95% CI = 0.25–0.42) after dichotomizing into a 0–1 score (Table 3).

**Table 3:** Agreement between STIR and T2W Dixon water only images for the initial assessment of areas with *increased bone marrow signal* in 77 healthy children and adolescents aged 6–19 years

		T2W Dixon intensity			Total
		No signal	Mild	Moderate-fluid	
STIR intensity	No signal	0	128	45	173
	Mild	94	78	24	196
	Moderate-fluid	47	38	91	176
Total		141	244	160	545

Absolute agreement was found for 169/545 (31.0%) lesions, with T2 Dixon-based reading identifying a 5.8% higher number of high signal areas than did STIR (*p* = 0.04). Collapsing scores 1 and 2 yielded a kappa value of –0.4 (95% CI = –0.36 to –0.44). The agreement did not differ according to age, with kappa values of –0.4 for all four age groups.

Extension of the high signal areas was scored for 185 of the 545 identified “true” high intensity signal areas. There was a moderate agreement between the T2W TSE Dixon and STIR-based scores on a 0–4 scale, with a kappa value of 0.45 (95% CI = 0.34–0.56) (Table 4). Excluding lesions with an extension below 5% did not improve the agreement (kappa value 0.45 (95% CI = 0.33–0.57)).

**Table 4:** Agreement between STIR and T2W Dixon water only images for the initial assessment of the *extension* of areas with increased bone marrow signal in 77 healthy children and adolescents aged 6–19 years

		T2W Dixon extension				Total
		< 5%	5%–1/3	1/3–2/3	2/3–3/3	
STIR extension	< 5%	30	33	1	1	65
	5%–1/3	5	88	10	2	105
	1/3–2/3	1	5	7	0	13
	2/3–3/3	0	0	0	2	2
Total		36	126	18	5	185

## Discussion

We have shown, in a large cohort of healthy children and adolescents, that 90% of high signal bone marrow areas identified on a 1.5-T whole-body MRI were seen on both STIR and water-only T2W Dixon, while 5% were seen on STIR only and 5% were seen on T2W Dixon only. Nearly all signal changes seen exclusively on one sequence had low conspicuity/signal intensity. T2W Dixon-based readings identified a 6% higher number of high signal areas, a higher proportion of true positives, and fewer false negatives than did STIR. Grading signal intensity on a 0–2 scale showed poor agreement; although this to some extent is due to inherent subjective reader variability, we believe it reflects that the two sequences display high signal areas differently.

The fact that nearly all areas of medium to fluid signal were visible on both scans when re-scored in consensus by three experienced radiologists (substitute gold standard) implies that high signal areas of clinical importance most likely will be identified on both scan types. Our results indicate that in follow-ups of known pathology in a particular patient, as well as when searching for multiplicity, the same protocol should be used. The higher sensitivity and specificity in identifying high signal areas, as well as the additional information provided by Dixon, favor this sequence over STIR, as does the fact that most of the images excluded for further analysis based on artifacts or suboptimal signal-to-noise ratio were STIR-weighted. The latter should be interpreted with caution, however, as the STIR sequence was performed at the end of the examination, with an increased risk of movement artifacts in a bored child.

Although the majority of the 545 “true” lesions were identified on both sequences, conspicuity, or signal intensity, was perceived and scored differently, with T2W Dixon identifying a slightly higher number of high signal areas. This might reflect differences between the two sequences beyond voxel size, image artifacts, and reading environments. We believe that the inconsistent initial grading as displayed in the contingency table (Table 3) is caused by the slightly lower SNR in STIR images (Fig. 2), small movements between scans (Fig. 4, Fig. 5), the subjective nature of the scoring, the large number of localizations assessed per whole-body MRI, or to a combination of these issues. Moreover, the choice of parameters might have played a role. Ideally, a comparison between the two sequences should have included a repeatability exercise for each of the two sequences, separately, to help identify, and thus exclude disagreement due to precision issues. However, based on 96 examinations from the same WB MRI study, we have previously shown that scoring high intensity bone marrow changes on a 0–2 scale performs well, both for the same, and between readers based on T2W Dixon images [7]. We assume that the precision of STIR-based readings does not differ substantially from the Dixon-based readings, an assumption supported by others [10]. Moreover, the technical settings used in our study were harmonized between the sequences. We therefore argue that a substantial part of the inconsistency seen between STIR and T2 Dixon-based readings of signal intensity is explained by true differences between the sequences.

In adults, several studies have compared the diagnostic performance of T2W Dixon and STIR sequences for focused examinations. In their study of 22 patients, mean age 80.9 years, Heynen et al. compared T2W Dixon, T1W, and STIR images in the assessment of radiographically occult fractures to the femoral neck, using a 3-T scanner [10]. Interestingly, the interobserver agreement was significantly lower for the T2W Dixon images than for the STIR images, with kappa values of 0.70–0.79 and 0.87–0.93, respectively. Moreover, using information for all available sequences as a substitute gold standard, T2W Dixon water-only images had lower sensitivity and accuracy for identification of fractures than had STIR [10]. Their results contrast those of others, for example in the assessment of the lumbar spine [11], the hand [5], and the sacro-iliac joints [12]. In sum, the published results vary significantly, reflecting different scanners and technical settings, as well as different study designs. Similar studies in children, or studies addressing whole-body MRI, do, to the best of our knowledge, not exist.

We found a moderate agreement between Dixon and STIR images for the assessment of signal extension, when based on 185 of the 545 high intensity areas. Again, the inconsistencies may be due to issues as listed for signal intensity, such as differences in SNR, small movements between scans, and the subjective nature of the scoring.

Again, we would argue that our results have implications for clinical practice, underscoring the importance of applying identical whole-body MRI protocols during follow-up of children with metastatic or inflammatory bone marrow lesions, particularly due to the high frequency of silent lesions [13, 14]. In our study, 5% of 545 lesions were visible on STIR only, and another 5% were visible on T2 Dixon only, based on a consensus score between three experienced readers. Similar differences most likely occur between MRI scanners from different vendors, sequences with different scan parameters, and between scanners of different field strengths. Compared to the 1.5-T scanner used in our study, a 3-T scanner has higher signal-to-noise ratio, wider chemical shift between the fat- and water-signal peak, higher specific absorption rate, larger susceptibility effects with resultant artifacts, greater B0 and B1 heterogeneity, and a longer T1 relaxation time. These differences affect the reliability and consistency of fat suppression, and, in theory after optimization of the technical settings, should provide better images, thus better observer agreement. In a busy environment, issues like these might be ignored due to availability constraints on MRI laboratories, among others.

We acknowledge several limitations to our study. First, our results apply to whole-body 1.5-T MRIs in healthy children only. However, a high proportion of the high signal intensity areas were grade 2, e.g., with a moderate to fluid like signal intensity, which in a clinical setting might mimic pathology [15, 16]. Second, subjective grading of signal intensity on fat-suppressed T2W images is hampered with difficulties; however, we have recently shown that grading intensity of high signal areas within the bone marrow performs well after meticulous calibration [7]. Third, blinding according to sequence was impossible and fourth, our choice of sequence parameters is only one of many options, and the conclusion holds for only these particular sequence parameters. The strengths include the meticulous calibration between observers prior to scoring, the relatively high numbers and balanced distribution of findings, and similar technical settings for both scans.

## Conclusion

We have shown, in a large cohort of healthy children and adolescents, that most high signal bone marrow changes identified on a 1.5-T whole-body MRI were seen on both STIR and water-only T2W Dixon, and that nearly all signal changes seen exclusively on one sequence had low conspicuity/signal intensity. T2W Dixon-based readings identified a 6% higher number of lesions, a higher proportion of true positives, and fewer false negatives than did STIR. This underscores the importance of using identical protocols when following bone-marrow signal changes over time. The additional information provided by the Dixon-w images, as well as the better SNR, however, favors the Dixon sequence.

## Data Availability

Data is available on direct request to the authors.

## Abbreviations

CHESS: Chemical shift–selective saturation
CI: Confidence interval
CNO: Chronic non-bacterial osteomyelitis
DWI: Diffusion-weighted images
FOPE: Periphyseal edema
LCH: Langerhans cell histiocytosis
SNR: Signal-to-noise ratio
SPIR: Spectral pre-saturation with inversion recovery
STIR: Short time inversion recovery
T1W: T1 weighted
T2W: T2 weighted
WBMRI: Whole-body magnetic resonance imaging

## Declarations

### Guarantor

The scientific guarantor of this publication is Karen Rosendahl.

### Statistics and biometry

No complex statistical methods were necessary for this paper.

### Study subjects or cohorts overlap

This paper is part of a large, multicentre prospective study, including WB MRIs of 196 healthy children and adolescents, aiming at (1) establishing, and validating a scoring system for high signal bone marrow changes; (2) examining the ability of different water-sensitive sequences to identify high signal bone marrow changes in healthy children; and (3) examining the prevalence, distribution and appearances of high signal bone marrow changes in healthy children and adolescents.

### Methodology

• prospective

• cross sectional study

• performed at one institution
